# High glucose promotes breast cancer proliferation and metastasis by impairing angiotensinogen expression

**DOI:** 10.1042/BSR20190436

**Published:** 2019-06-14

**Authors:** Shichao Sun, Yao Sun, Xiaoping Rong, Lei Bai

**Affiliations:** 1 Department of Neurology, the Second Hospital, Hebei Medical University, No. 215 Heping West Road, Xinhua District, Shijiazhuang 050000, Hebei, China; 2 Department of Medical Image, the Fourth Hospital, Hebei Medical University, No. 12 Jiankang Road, Shijiazhuang 050011, Hebei, China; 3 Department of Pediatrics, the Fourth Hospital, Hebei Medical University, No. 12 Jiankang Road, Shijiazhuang 050011, Hebei, China; 4 Department of Endocrinology, the Fourth Hospital, Hebei Medical University, No. 12 Jiankang Road, Shijiazhuang 050011, Hebei, China

**Keywords:** angiotensinogen, breast cancer, high glucose, proliferation

## Abstract

A number of investigations have addressed the importance of high glucose in breast cancer, however, the involvement of angiotensinogen (AGT) in this scenario is yet to be defined. Here we set out to analyze the potential pro-tumor effects of high glucose in breast cancer, and understand the underlying molecular mechanism. We demonstrated that high glucose promoted cell proliferation, viability, and anchorage-independent growth of breast cancer cells. In addition, the migrative and invasive capacities were significantly enhanced by high glucose medium. Mechanistically, AGT expression was inhibited by high glucose at both transcriptional and translational levels. High AGT remarkably suppressed proliferation, inhibited viability, and compromised migration/invasion of breast cancer cells. Most importantly, ectopic introduction of AGT almost completely abrogated pro-tumor effects of high glucose. Our study has characterized the pro-tumor properties of high glucose in breast cancer cells, which is predominantly attributed to the suppression of AGT.

## Introduction

Breast cancer is one of the most common gynecological malignancies, which accounts for 25% of all incidences. In 2012, it was estimated that 1.68 million new patients were diagnosed and 522000 cancer-related deaths were claimed [1]. Epidemiological investigation revealed the commonality of breast cancer in developed countries in comparison with developing ones. Regarding gender influences, it was reported that the morbidity of breast cancer is 100 times higher in women than men. The recognized risk factors associated with breast cancer include gender, obesity, less physical exercise, excessive alcohol consumption, hormone supplementation therapy during mesopause, radiation exposure, old age, and genetic pre-disposition [2]. Approximately 5–10% breast cancer link to hereditary genetic aberrances such as BRCA1 and BRCA2 [3]. Histologically, most breast cancers are categorized into ductal carcinoma and lobular carcinoma based on cellular origins. The diagnosis of breast cancer is usually performed by imaging and confirmed by biopsy of the concerning lump. Beneficial effects of regular screening of breast cancer are controversial so far regarding the inconsistent conclusions from different investigations [4]. Preventive therapies are sometimes applicable to those population with high risk to develop breast cancer, including medications such as tamoxifen and raloxifene and surgical removal of both breasts. Clinical managements of this disease depend on cancer type, extent of disease, and individual health conditions, as well as surgery, radiation therapy, chemotherapy, hormonal therapy, and targeted therapy [5]. Prognosis of this disease varies and overall 5-year survival rates are approximately 80–90% in the U.S.A.

Diabetes refers to a group of metabolic disorders which feature high blood sugar levels over a prolonged period and symptomatic frequent urination, increased thirst, and increased hunger [6]. Epidemiologic studies suggested that diabetic patients were at significant higher risk for a number of cancers including pancreas, endometrium, liver, colon/rectum, bladder, and breast cancers [7]. For instance, a prospective study performed by Jee et al. in a Korean cohort aged 30–95 years old disclosed elevated cancer incidence for patients with diabetes mellitus or high blood glucose level (>125 mg/dl), in comparison with hyperglycemic ones [8]. Ben et al. provided evidences in support of the intimate association between diabetes and pancreatic cancer with risk factor approximately 1.94, wherein the highest risk was observed in those patients diagnosed within one year [9]. Campbell et al. uncovered the association between colorectal cancer and type 2 diabetes mellitus or insulin use in men [10]. El-Serag et al. provided evidences in support of the association between diabetes and hepatocellular carcinoma through epidemiologic evidence [11]. Regarding breast cancer, Michels et al. displayed type 2 diabetes and subsequent incidence of breast cancer in the Nurses’ Health Study [12], which was consolidated by the concomitant meta-analysis performed by Larsson et al. [13] and Wolf et al. [14]. Consistent with the clinical observations, an array of *in vitro* evidences addressed the potential impacts of high glucose on tumor biology of breast cancer. Flores-Lopez et al. showed high glucose and insulin enhanced uPA expression, invasiveness and ROS formation in breast cancer-derived cells [15]. Takatani-Nakase et al. demonstrated that high glucose level promoted migrative behavior of breast cancer cells via zinc and its transporters [16]. Wei et al. proposed that high glucose and high insulin conditions promoted MCF-7 cell proliferation and invasion by upregulating IRS1 and activating the Ras/Raf/ERK pathway [17]. The above-mentioned results indicated the diverse mode-of-action underlying the contributions of high glucose to breast cancer, which prompted us to investigate this phenotype while focusing on the possible negative regulation on potential tumor suppressors.

## Materials and methods

### Cell culture

Human breast cancer cell lines MCF-7 and MDA-MB-231 were received from the American Type Culture Collection (VA, U.S.A.) and grown in RPMI-1640 medium (Hyclone, ThermoFisher, MA, U.S.A.) containing 10% fetal bovine serum (FBS, HyClone) and 1% penicillin/streptomycin (Gibco, MA, U.S.A.). Cells culture was maintained in humidified CO_2_ (5%) incubator. Cell transfection was performed with DharmaFECT Transfection Reagents (ThermoFisher, MA, U.S.A.) according to the manufacturer’s instruction.

### 3-(4,5-Dimethylthiazol-2-yl)-2,5-diphenyltetrazolium bromide (MTT) assay

To generate viability curves, 500 cells/100 μl were seeded into 96-well plate and cultured in regular medium for 24 h. The medium was replaced by MTT reagent (Sigma, MO, U.S.A.) dissolved in phosphate buffered saline and incubated for 3 h at 37°C. The resultant formazan was dissolved by 200 μl of dimethyl sulfoxide and absorbance at 570 nm was measured with ELx800 microplate reader (BioTek Instruments, VT, U.S.A.).

### Cell counting

The indicated cells were plated into 6-well plate at a density of 5 × 10^4^ cells/2 ml and grown for 72 h. Cells were harvested and digested into single-cell solution and subjected to trypan blue staining. The viable cells were counted under the light microscope using hemocytometer.

### Transwell assay

Transwell chambers (Corning, NY, U.S.A.) were used to measure migrative capacity. Indicated cells (5 × 10^5^ cells/well in serum-free medium) were plated in the upper chamber. The lower compartment was filled with 0.75 ml complete medium plus extra 5% FBS. Cells were cultured for 10 h at 37°C in CO_2_ incubator. Non-migrated cells were carefully swiped with cotton swabs. Cells on the polycarbonate membrane were stained with 0.2% crystal violet. Cells were counted in five random fields to calculate the migration. For cell invasive assay, the chamber was pre-coated with Matrigel (0.25 mg/ml; BD Biosciences, CA, U.S.A.) and followed by the same procedure as described above.

### Soft agar assay

The 6-well plate was pre-coated with 0.75% agarose. The single-cell suspension was prepared in 0.25% low melting agarose (2 × 10^4^ cells/ml) and cautiously laid onto the supporting layer. Cells were cultured for 3 weeks in the CO_2_ chamber. The formed colonies were stained and counted under a light microscope (Olympus, Tokyo, Japan).

### Western blot

Cells were harvested and lysed in radioimmunoprecipitation assay buffer (Beyotime, Nantong, China). Equal amounts of proteins were resolved by 12% sodium dodecyl sulfate polyacrylamide gel electrophoresis and transferred onto methanol pre-activated polyvinylidene difluoride membrane on ice. 5% non-fat milk was used for blocking purpose. Incubation with specific primary antibody [anti-Angiotensinogen (AGT), #79299, 1:1000; anti-actin, #4967, 1:1000, Cell Signaling Technology, MA, U.S.A.] was performed at 4°C overnight and followed by horseradish peroxidase-conjugated secondary antibody (anti-rabbit, #7074, 1:5000, Cell Signaling Technology, MA, U.S.A.) hybridization. The bands were visualized by ECL (Millipore, CA, U.S.A.).

### Real-time polymerase chain reaction

Total RNA extraction was performed with BiooPure RNA Isolation Reagent (Bioo Scientific, TX, U.S.A.). Reverse transcription was prepared by the RevertAid First Strand cDNA Synthesis Kit (Fermentas, MD, U.S.A.). Real-time polymerase chain reaction (PCR) was employed the SYBR Green MasterMix (Applied BioSystems, CA, U.S.A.) and followed the manufacturer’s instructions. Relative quantitation was calculated by the 2-∆∆Ct method and normalized to β-actin. The primer sequences were provided as follows:

AGT forward, 5′-CCCCAGTCTGAGATGGCTC-3′

AGT reverse, 5′-GACGAGGTGGAAGGGGTGTA-3′

β-actin forward, 5′-ACAGAGCCTCGCCTTTGCCGAT-3′

β-actin reverse, 5′-CTTGCACATGCCGGAGCCGTT-3′

### Statistical analysis

Statistical analysis was performed using SPSS 22.0. Statistical comparison was analyzed by Student’s *t*-test or one- or two-way ANOVA followed by a post hoc test. *P*-values less than 0.05 were considered significantly different. All results were acquired from at least three independent experiments.

## Results

### The influence of high glucose on cell proliferation

**Figure 1:**
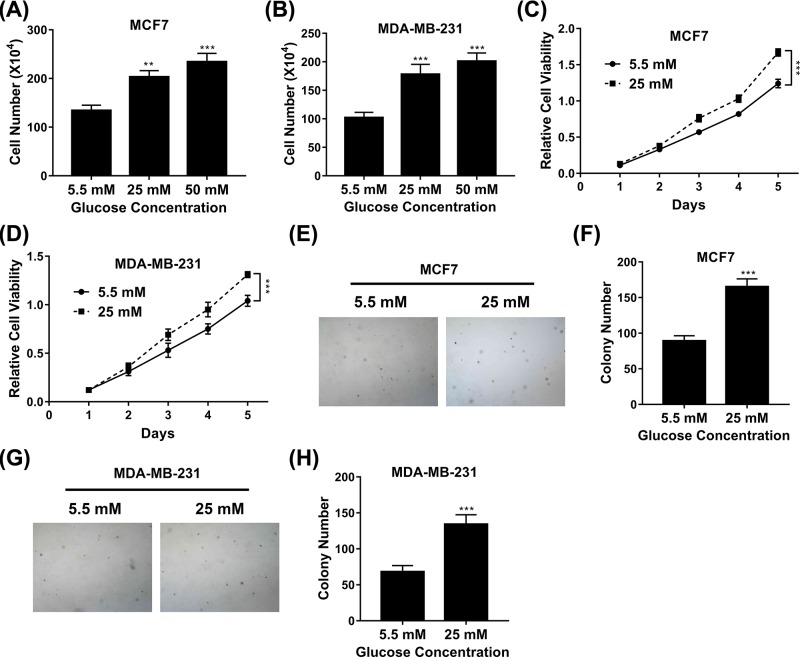
Influence of high glucose on cell proliferation (**A,B**) Proliferation of MCF7 (**A**) and MDA-MB-231 (**B**) cells exposed to medium with glucose concentrations varying from 5.5 to 50 mM was determined by cell counting assay. (**C,D**) Proliferation of MCF7 (**C**) and MDA-MB-231 (**D**) cells exposed to medium with glucose concentrations of 5.5 or 25 mM was determined by MTT assay. (**E,F**) Proliferation of MCF7 cells exposed to medium with glucose concentrations of 5.5 or 25 mM was determined by soft agar assay. (**G,H**) Proliferation of MDA-MB-231 cells exposed to medium with glucose concentrations of 5.5 or 25 mM was determined by soft agar assay. Data are shown as mean ± S.D. **P<0.01; ***P<0.001; (ANOVA test in figures C and D, others Student’s *t*-test).

We first set out to evaluate the potential influence of high glucose on proliferative index in breast cancer cells. Cell proliferation was determined by cell counting under normal culture conditions with different concentration of glucose ranging from 5.5, 25 to 50 mM. Our data clearly showed that supplementation with 25 mM of glucose significantly promoted proliferation of both MCF-7 and MDA-MB-231 cells, in comparison with 5.5 mM glucose in the normal culture medium (Figure 1A,B), which implicated a critical role of high blood glucose in the tumor biology of breast cancer. We further confirmed this phenotype in both cell lines using the MTT assay. As shown in Figure 1C,D, cell viability was measured at different time points upon high glucose treatments, and the result was definitely in support of the pro-proliferative effect of high glucose on cell growth. The anchorage-independent growth of breast cancer cells was analyzed by soft agar assay as well. The colony number of MCF-7 and MDA-MB was evidently increased in high glucose medium in comparison with low glucose counterparts (Figure 1E–H), and the representative images were provided. Our results unambiguously suggested that high glucose critically contributed to the growth of breast cancer cells.

### The influences of high glucose on cell migration and invasion

**Figure 2:**
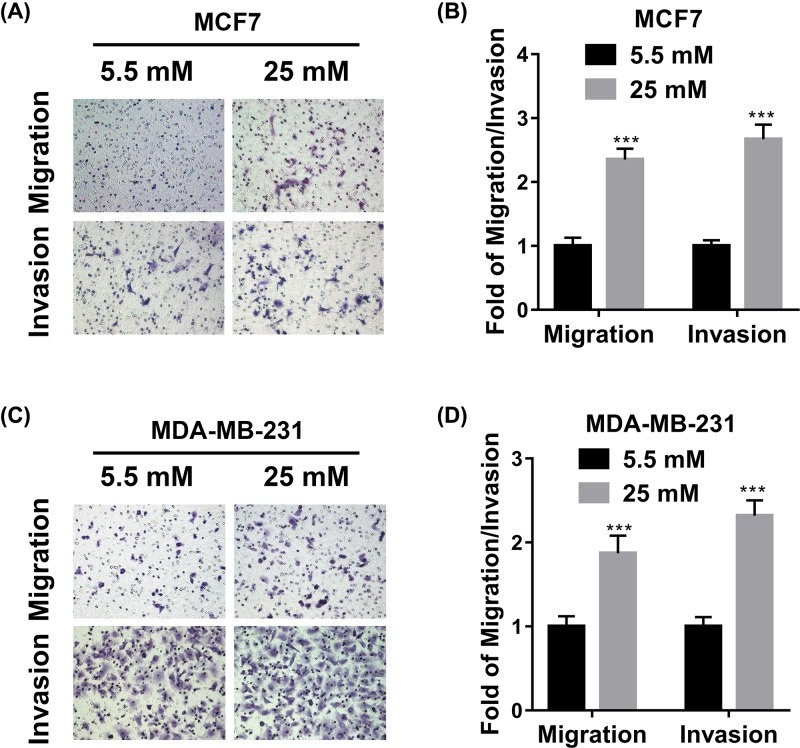
Influence of high glucose on cell migration and invasion (**A,C**) Transwell migration and invasion assays of MCF7 (**A**) and MDA-MB-231 (**C**) cells exposed to medium with glucose concentrations of 5.5 or 25 mM. (**B,D**) The statistical results of transwell migration and invasion assays in (**A,C**). Data are shown as mean ± S.D. ***P<0.001; (Student’s *t*-test).

Next, we sought to investigate possible impacts of high glucose on malignant metastatic behavior in breast cancer cell lines. To this end, we employed the transwell chambers to measure cell migration and Matrigel-precoated transwell to measure cell invasion capacity, respectively. The presence of high glucose in the attractant medium significantly stimulated cell migration of both MCF-7 and MDA-MB-231 cells (Figure 2A,B). Likewise, the invasive capacities of both cell lines were greatly improved by supplementation with high concentration of glucose (Figure 2C,D). Our data suggested that high glucose promoted cell migration and invasion *in vitro* in addition to its pro-proliferative effects.

### High glucose inhibits AGT expression

**Figure 3:**
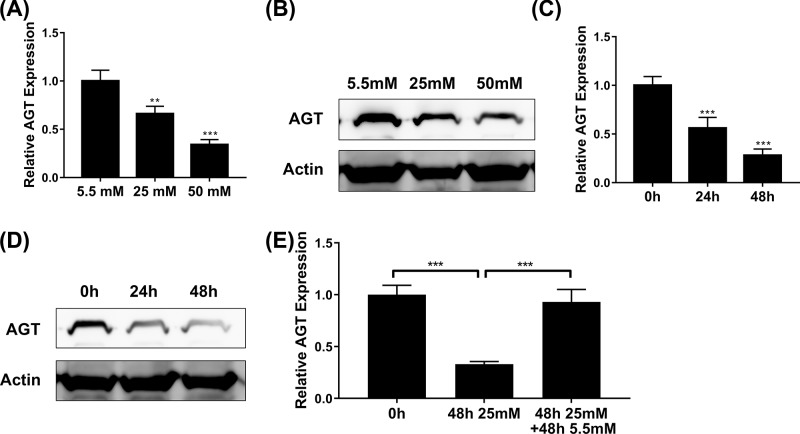
High glucose inhibits AGT expression (**A,B**) The expression of AGT in MCF7 cells exposed to medium with glucose concentrations of 5.5, 25, or 50 mM was determined by qPCR (**A**) and Western blot (**B**). (**C,D**) The expression of AGT in MCF7 cells exposed to medium with glucose concentrations 25 mM for 24 or 48 h was determined by qPCR (**C**) and Western blot (**D**). (**E**) The expression of AGT in MCF7 cells exposed to medium with glucose concentrations 25 mM for 48 h and then removed with 5.5 mM for 48 h was determined by qPCR. Data are shown as mean ± S.D. **P<0.01; ***P<0.001; (ANOVA).

Our previous results uncovered the pro-tumoral properties of high glucose in breast cancer cells. Next, we sought to understand the underlying molecular mechanisms in this scenario. We focused on AGT in view of its sensor role to the blood glucose and the recognized tumor suppressor function in hepatocellular carcinoma. The relative expression of AGT was determined at both transcript and protein levels by real-time PCR and Western blot, respectively. As shown in Figure 3A,B, AGT expression was remarkably suppressed by high glucose in a dose-dependent manner, which indicated an unappreciated pathway through which high glucose inhibited AGT transcription. Similarly, the time course of AGT expression alteration was measured upon the presence of high level glucose in the culture medium, wherein both transcript and protein of AGT were reduced over time (Figure 3C,D). Replacement with low glucose in the culture medium for 48 h completely restored the suppressed expression of AGT under high glucose conditions (Figure 3E). Therefore, we, for the first time, disclosed that transcription of AGT in breast cancer cells was subjected to the negative regulatory action of glucose concentration, which might consequently underlie the pro-tumoral activities of high glucose in breast cancer cells.

### AGT inhibits breast cancer proliferation and metastasis

**Figure 4:**
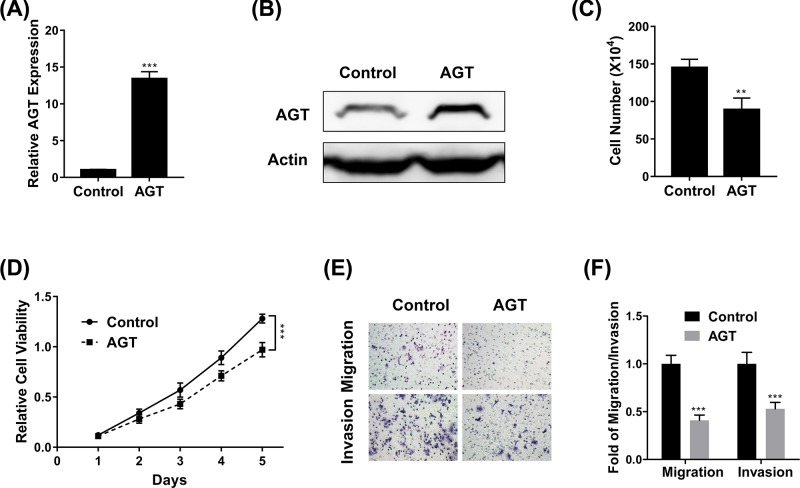
AGT inhibits breast cancer proliferation and metastasis (**A,B**) The expression of AGT in MCF7 cells transfected with AGT overexpression plasmid was determined by qPCR (**A**) and Western blot (**B**). (**C,D**) Proliferation of MCF7 cells transfected with AGT expression plasmid was determined by cell counting (**C**) and MTT (**D**) assays. (**E,F**) Transwell migration and invasion assays of MCF7 cells transfected with AGT expression plasmid. Data are shown as mean ± S.D. **P<0.01; ***P<0.001; (ANOVA test in figure D, others Student’s *t*-test).

As mentioned before, the tumor suppressor property of AGT has been identified in hepatocellular carcinoma by Vincent et al. [18], which prompted us to experimentally validate the similar effects in breast cancer cells. To this purpose, we first established stable cell line with ectopic over-expression of AGT in MCF-7. The successful establishment of AGT-proficient cell line was confirmed by both real-time PCR (Figure 4A) and Western blot (Figure 4B). We then assessed the alterations in terms of cell proliferation, viability, migration, and invasion in response to AGT overexpression. Under normal culture conditions, cell proliferation was significantly suppressed in AGT-proficient MCF-7 cells in comparison with the control (Figure 4C). Consistent with the inhibitory effect of AGT on cell growth, our MTT results further provided evidence that cell viability was compromised by AGT as well (Figure 4D). In contrast with the pro-metastatic activities of high glucose, we demonstrated that forced over-expression of AGT in MCF-7 cells remarkably inhibited both cell migration (Figure 4E) and invasion (Figure 4F). Our results characterized the anti-tumor properties of AGT in breast cancer cells, which might eventually underlie the pro-tumoral effects of high glucose in this disease.

### Overexpression of AGT inhibits the influence of high glucose on cell proliferation and metastasis

**Figure 5:**
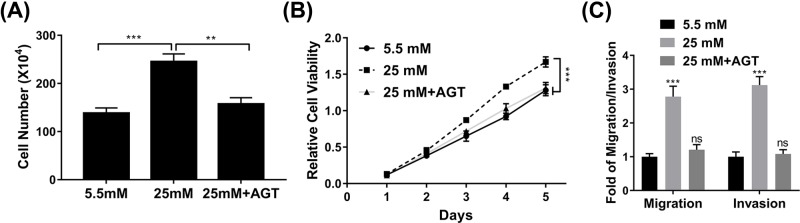
Overexpression of AGT inhibits the effect of high glucose on cell proliferation and metastasis (**A,B**) Proliferation of MCF7 cells exposed to medium with glucose concentrations of 25 mM and/or AGT expression plasmid was determined by cell counting (**A**) and MTT (**B**) assays. (**C**) Transwell migration and invasion assay of MCF7 cells exposed to medium with glucose concentrations of 25 mM and/or AGT expression plasmid. Data are shown as mean ± S.D. **P<0.01; ***P<0.001; ns, not significant (ANOVA).

Our previous finding validated the tumor suppressor role of AGT in breast cancer cells, which prompted us to clarify whether suppression of AGT elicited by high glucose treatment predominantly contributed to its pro-tumoral effects. To this end, we challenged AGT-proficient MCF-7 cells with high concentration of glucose, and impacted malignant indexes were examined. As shown in Figure 5A, the observed significant increase in terms of cell proliferation stimulated by high glucose treatment was completely reversed by co-overexpression of AGT. Likewise, cell viability was suppressed in AGT-expressing MCF-7 cells in comparison with wild-type cells when exposed to high dose of glucose (Figure 5B). Consistently, both cell migration and invasion were inhibited by forced expression of AGT in MCF-7 cells in response to glucose treatment (Figure 5C). Our data clearly highlighted the critical roles of AGT in mediating the pro-tumoral properties of high glucose in breast cancer cells.

## Discussion

The coincidence of breast cancer and diabetes has been increasingly recognized clinically. Accumulative evidences have unraveled the intimate association between high blood glucose and a variety of human malignancies. Here we set out to clarify the potential influences of high glucose concentration on tumor biology of breast cancer *in vitro*, and attempted to elucidate the underlying mechanism. Our data clearly demonstrated that high glucose promoted cell proliferation, viability, and anchorage-independent growth. In addition, cell migration and invasion capacity were significantly enhanced in the high glucose medium in comparison with normal culture medium, which indicated that high glucose contributed to the metastatic behavior of breast cancer as well. Mechanistically, we characterized the remarkable down-regulation of AGT by high glucose in MCF-7 cells at both transcriptional and translational levels in a dose-dependent manner. Consistent with previously reported tumor suppressor function, here we showed that ectopic overexpression of AGT in MCF-7 cells significantly suppressed cell proliferation, anchorage-independent colony formation, migration, and invasion. Most notably, exogenously introduced AGT almost completely abrogated the high glucose-elicited pro-proliferative and pro-metastatic effects on MCF-7 cells, which indicated that the pro-tumor properties of high glucose were heavily depended on suppression of AGT expression. Therefore, our study unambiguously demonstrated the oncogenic contributions of high glucose in breast cancer cells in terms of growth and metastasis, which was predominantly mediated by the suppressive expression of AGT. Our data underlined the critical role of AGT in the tumor biology of breast cancer, which was responded to and down-regulated by high glucose and consequently contributed to the malignant proliferation and metastasis.

The susceptibility of AGT expression to glucose concentration has been investigated in various scenarios so far. For instance, Wang et al. reported that high glucose augmented AGT in human kidney proximal tubular cells via hepatocyte nuclear factor-5 [17]. Peng et al. suggested that high glucose stimulated the activation of local renin-angiotensin system in glomerular endothelial cells, which was further indicated to be involved in albumin permeability in glomerular endothelial cells under high glucose exposure [19]. Zhang et al. reported that high levels of glucose stimulated AGT gene expression via the p38/MAPK signaling pathway in rat kidney proximal tubular cells [20]. Deb et al. showed that 1,25-dihydroxyvitamin D3 suppressed high glucose-induced AGT expression in kidney cells by blocking the NF-κB pathway [21]. Hsieh et al. proposed that high glucose stimulated AGT gene expression and cell hypertrophy via either activating the hexosamine biosynthesis pathway or reactive oxygen species generation in rat kidney proximal tubular cells [22]. In contrast with all above-mentioned observations, here we provided evidences that AGT was significantly inhibited at both transcriptional and translational levels in breast cancer cells by high glucose in culture medium. Most notably, we unraveled the tumor suppressor role of AGT in breast cancer through suppression of cell proliferation and inhibition of metastasis-related migration and invasion behaviors, and forced overexpression of AGT completely abolished the pro-tumor actions of high glucose in breast cancer, which highlighted the critical role of AGT underlying this phenotype. Therefore, we proposed that aberrant down-regulation of AGT served as a prognostic biomarker for breast cancer, and restoration of AGT expression might be a promising therapeutic strategy in consideration of the high blood glucoses exposure of this disease. Noting worthily, multipole investigations suggested the biomarker value of AGT polymorphisms in breast cancer as well, which implicated potential alterations in terms of expression or activity [23–25].

The mechanistic involvements of the dysregulated AGT have been documented in a number of human malignancies. Wang et al. uncovered the genetic association between AGT polymorphisms and the risk in lung cancer [26]. Urup et al. demonstrated that AGT and HLA subclass II predicted bevacizumab response in recurrent glioblastoma patients [27]. The T174M polymorphism in the AGT gene was further identified to associate with the risk of myocardial infarction in a meta-analysis study [28]. Shimomoto et al. showed that diabetes-associated angiotensin activation enhanced liver metastasis of colon cancer [29]. Luo et al. demonstrated that anti-angiotensin and hypoglycemic treatments suppressed liver metastasis of colon cancer cells [30]. The proof-of-concept investigation performed by Bouquet et al. reported that adenovirus-mediated gene transfer of human AGT suppressed angiogenesis, tumor growth, and metastasis in breast cancer [31]. The investigation presented by Chai et al. proposed the potential prognostic value of AGT along with other serum proteins for lymph node metastasis in oral cancer [32]. In line with the acknowledged anti-tumor role of AGT, here we demonstrated that AGT was subjected to negative regulation by glucose concentration, and supplementation with ectopic expression of AGT significantly abrogated pro-tumor effects imposed by high glucose. It is worth noting that the regulatory mechanism underlying the suppressive expression of AGT in response to high glucose is still elusive. Furthermore, the mode-of-action of AGT played as tumor suppressor in the context of high glucose is still to be defined. It is highly likely that several elementary signaling pathways might contribute to this phenotype. The FAK and RhoA/Cdc42 pathway was reported to mediate Ang II-AT2R increased mesenchymal stem cell migration *in vitro* [33]. NOXs and MAPK pathways have been proposed to function in the early and late inflammatory responses induced by Ang II [34]. In diabetic renal dysfunction in rodents and humans, Gagliardini et al. unraveled that Ang II played critical role via regulation of Notch/Snail pathway [35]. In view of well-acknowledged importance of these signaling cues in human cancers, whether they were involved in this scenario definitely worthied further investigations.

## Conclusion

In summary, our study uncovered the pro-tumor properties of high glucose, which might contribute to the coincidence between diabetes and breast cancer. We also unraveled that the suppressed expression of AGT predominantly mediated this effect.

## Abbreviations

AGT: angiotensinogen
MTT: 3-(4,5-dimethylthiazol-2-yl)-2,5-diphenyltetrazolium bromide
